# ULK2 Ser 1027 Phosphorylation by PKA Regulates Its Nuclear Localization Occurring through Karyopherin Beta 2 Recognition of a PY-NLS Motif

**DOI:** 10.1371/journal.pone.0127784

**Published:** 2015-06-08

**Authors:** Sung Hwa Shin, Eun Jeoung Lee, Jaesun Chun, Sunghee Hyun, Sang Sun Kang

**Affiliations:** 1 Department of Biology Education, Chungbuk National University, 1, Chungdae-ro, Seowon-gu, Cheongju, Chungbuk, Republic of Korea; 2 Department of Internal Medicine, Chungbuk National University, 1, Chungdae-ro, Seowon-gu, Cheongju, Chungbuk, Republic of Korea; 3 Department of Biology Education, Korea National University of Education, Taeseongtabyeon-ro, Heungdeok-gu, Cheongju, Chungbuk, Republic of Korea; 4 Department of Biomedical Laboratory Science, Eulji University, Daejeon, Republic of Korea; Institute of Enzymology of the Hungarian Academy of Science, HUNGARY

## Abstract

Uncoordinated 51-like kinase 2 (ULK2), a member of the serine/threonine kinase family, plays an essential role in the regulation of autophagy in mammalian cells. Given the role of autophagy in normal cellular homeostasis and in multiple diseases, improved mechanistic insight into this process may result in the development of novel therapeutic approaches. Here, we present evidence that ULK2 associates with karyopherin beta 2 (Kapβ2) for its transportation into the nucleus. We identify a potential PY-NLS motif (^774^gpgfgssppGaeaapslRyvPY^795^) in the S/P space domain of ULK2, which is similar to the consensus PY-NLS motif (R/K/H)*X*_2–5_PY. Using a pull-down approach, we observe that ULK2 interacts physically with Kapβ2 both *in vitro and in vivo*. Confocal microscopy confirmed the co-localization of ULK2 and Kapβ2. Localization of ULK2 to the nuclear region was disrupted by mutations in the putative Kapβ2-binding motif (P794A). Furthermore, in transient transfection assays, the presence of the Kapβ2 binding site mutant (the cytoplasmic localization form) was associated with a substantial increase in autophagy activity (but a decrease in the *in vitro* serine-phosphorylation) compared with the wild type ULK2. Mutational analysis showed that the phosphorylation on the Ser1027 residue of ULK2 by Protein Kinase A (PKA) is the regulatory point for its functional dissociation from Atg13 and FIP 200, nuclear localization, and autophagy. Taken together, our observations indicate that Kapβ2 interacts with ULK2 through ULK2’s putative PY-NLS motif, and facilitates transport from the cytoplasm to the nucleus, depending on its Ser1027 residue phosphorylation by PKA, thereby reducing autophagic activity.

## Introduction

Uncoordinated 51-like kinase 2 (ULK2) is a member of the serine/threonine kinase protein family, which plays an essential role in the regulation of autophagy in mammalian cells [1]. Similar to ULK1, ULK2 is expressed ubiquitously, and its function appears to be redundant with that of ULK1 [2,3], since ULK2 can compensate for the deletion of ULK1. Due to this phenomenon, the specific roles of ULK1 and ULK2 in autophagy are not yet clear [1,4,5].

The central role of autophagy in normal cellular homeostasis and multiple diseases suggests that mechanistic insights into autophagy could drive the development of novel therapeutic approaches [6–8]. Few enzymes exert as broad a regulatory influence on cellular function as does ULK2 [1,4,5,7], which is involved in many fundamental biological processes, including cell fate determination, metabolism, transcriptional control, and oncogenesis [7,9]. Similar to other ULK family members, ULK2 also plays a central role in the autophagy signaling pathway [1–9]. Recently, others have suggested that the activity of ULK2 must be carefully regulated by mechanisms that are individually tailored to each substrate in order to avoid indiscriminate phosphorylation by ULK1 [2–5].

Although the mechanisms that regulate ULK2 in the autophagic process are not yet fully understood, precise control appears to be achieved through a combination of phosphorylation, localization, and interactions with ULK2 binding proteins [10]. Unlike ULK1, which is predominantly found in the cytosol, ULK2 is located mainly in the nucleus, but can also be found in the cytosol and mitochondria [1–5]. However, the mechanism by which ULK2 is localized to the nucleus has not yet been determined since ULK2 does not have any recognizable short, basic, classic import or export sequences [1,3,5]. Thus, localization is likely indirectly regulated through association with binding proteins, and it has been suggested that a binding protein may regulate the subcellular localization of ULK2 by inhibiting its nuclear export.

A related family of shuttling transport factors, importins and exportins, recognizes nuclear localization sequence (NLS)-containing or nuclear export sequence-containing proteins, and coordinates trafficking between the nucleus and the cytoplasm [11–13]. Kapβ2 (importin 2) has been identified as an import receptor that directly recognizes PY-NLS sequences, and is responsible for the import of PY-NLS-containing proteins [14,15]. It has been proposed that the nuclear localization of PY-NLS-containing proteins is mediated by their N-termini and the binding partner, Ran-GTP, and that other Kapβ2 sequences provide a docking site for PY-NLS motif-containing proteins [11,15]. PY-NLS is a relatively small, well-defined NLS that has concentrated binding energy. Structural and biochemical studies of Kapβ2 have revealed that the PY-NLS motif of its substrate proteins comprises a N-terminal hydrophobic or basic motif and a C-terminal (R/K/H)*X*_2–5_PY motif that Kapβ2 recognizes [11,14–16].

Here, we identify a putative PY-NLS motif in ULK2, and demonstrate that ULK2 interacts with Kapβ2, resulting in ULK2 nuclear localization. Upon visual inspection of the amino acid sequence of ULK2 for a RX_2–5_PY motif, we identified a potential Kapβ2 binding motif (^220^qdlrmfyeKnRslmpSipRetsPY^243^) within the C-terminus of the kinase domain, and another potential motif (^774^gpGfgssppgaeaapslRyvPY^795^) in the S/P space domain [1,3,5]. Consequently, we set out to determine whether ULK2 does in fact interact with Kapβ2. Our results demonstrate that Kapβ2 does interact with wild type ULK2 through ULK2’s putative PY-NLS motif (^774^gp**g**fgssppgaeaapslRyvPY^795^) in the S/P space domain, and to a lesser extent through the motif (^220^qdlrmfyeKnRslmp**s**ipRetsPY^243^) in the kinase domain. In addition, we provide evidence that ULK2’s interaction with Kapβ2 mediates the subcellular localization of ULK2, and also leads to downregulation of its autophagic activity. Furthermore, the (de)phosphorylation of the ULK2 Ser1027 residue (^1024^RRlSA^1028^) in its C-terminal domain (CTD) is the regulatory point of its functional association with Atg13 and FIP 200, nuclear localization, autophagy, and apoptosis. Thus, our observations provide insight into the molecular mechanisms underlying ULK2 regulation, activation, and nuclear import/export by Protein Kinase A (PKA) phosphorylation.

## Materials and Methods

### Reagents

Protease inhibitor cocktail was obtained from Roche Molecular Biochemicals in tablet form. Monoclonal and polyclonal antibodies against Kapβ2, ULK1, ULK2, LC3-II, Atg13, FIP200, WIPI, and phosphor Ser were purchased from Abcam (UK) or Cell Signaling (Boston, MA, USA).

### Cell culture and transfections

HEK293 cells were cultured in DMEM supplemented with 10% (v/v) heat-inactivated fetal bovine serum (FBS) and 1000U penicillin-streptomycin (Gibco BRL). HEK293 cells were maintained in DMEM supplemented with 10% (v/v) FBS and antibiotics. Transfections were conducted with Lipofectamine and Plus reagents (Invitrogen) in accordance with the manufacturer's instructions.

### Plasmid constructs

Human ULK2 cDNA was obtained from the Korean Human Gene Bank (Gene ID; KIAA0623), and cloned into the pEGFP C2 vector (Clontech, Palo Alto, CA, USA; EGFP was added to the C-terminus of ULK2) using the following primers: forward, 5’-CTCAAGCTTCGAATTCATGGAGGTGGTGGGTGAC-3’ and reverse 5’- CATAGCACCGCAACCGTGTGAGAATTCTGCAGTCGAC-3’. In order to generate karyopherin beta 2-binding motif mutants, ULK2 was subjected to site-directed mutagenesis using the following mutagenic primers: forward 5'-CCC AGA GAA ACA TCA GCT TAT TTG GCT AAT CTC-3' and reverse 5'-ATT AGC CAA ATA AGC TGA TGT TTC TCT GGG AAT-3'; forward 5'-AGC CTG AGA TAC GCT TAC GGT TGT TCT TGT-3' and reverse 5'-TGA AGC ACC GTA AGC CAC GTA TCT CAG GCT GGG-3'. For the generation of S1027A and S1027D mutants, ULK2 was subjected to site-directed mutagenesis using the following mutagenic primers: forward 5'-ATT GAG AGA AGA CTG GCG GCG CTC TGC CAT AGC-3' and reverse 5'-ATG GCA GAG CGC CGC CAG TCT TCT CTC AAT ACT-3'; forward 5'-ATT GAG AGA AGA CTG GAC GCG CTC TGC CAT AGC-3' and reverse 5'-ATG GCA GAG CGC GTC CAG TCT TCT CTC AAT ACT-3' using a QuikChange Multi Mutagenesis Kit (Stratagene, West Cedar, TX, USA) according to the manufacturer's instructions. GST-ULK2 WT (1–600 aa) or (601–1036 aa), containing a potential Kapβ2 binding motif (^220^qdlrmfyeKnRslmpSipRetsPY^243^) in the protein kinase domain or a potential Kapβ2 binding motif (^774^gpGfgssppgaeaapslRyvPY^795^) in the S/P space domain, were constructed with the following primers: forward, 5'- GGTTAA C CCG GG A ATG GAG GTG GTG GGT GAC GAC TTC-3’ and 5'- G GTT CTC GAG AGA GCC AAT GAT TGT TGG CAA AGG– 3’; and forward 5'- GGTTAA C CCG GG A ATGCCT ACT AAGACCACAGCT– 3’ and reverse 5'- GGTT CTC GAG TCACACGGTTGCGGTGCTATGGCA– 3’), respectively. PCR products were digested with SmaI and XhoI, and cloned into the pGEX-5X-1 vector. All GST-ULK2 fusion constructs were confirmed by DNA sequencing. GST-tagged recombinant ULK2 or Kapβ2 protein expressed in Escherichia coli BL21 (DE3) cells was purified with glutathione-agarose beads according to the manufacturer’s instructions (Amersham Biosciences, CA, USA). Purified proteins were used for pull-down assays.

### Immunoprecipitation

Cells were routinely analyzed 48 hours post-transfection. Cells were rinsed with ice-cold phosphate-buffered saline and resuspended in 1ml extraction buffer [10mM Tris-HCl pH7.4, 1mM EDTA, 5mM DTT, 100mM NaCl, 1.0% Triton X-100, 60mM n-octylglucoside, 1mM vanadate, 100μM molybdate, 20mM sodium fluoride and protease inhibitor cocktail (1 tablet per 10ml extraction buffer)]. The pre-cleaned lysate was incubated for 1 hour at 4°C with the appropriate antibody, and the resulting immune complexes were collected using Protein A-Sepharose beads (Pharmacia Co., NJ, USA). Immune complexes were then captured by centrifugation, washed extensively in lysis buffer, and solubilized with 2x sample buffer prior to loading onto a 10% SDS-PAGE gel.

### ULK2 or Kapβ2 pull-down assay

Whole cell lysates of HEK293 cells transiently expressing ULK2 were pre-cleaned with glutathione agarose beads, and 1μg of each glutathione agarose-tagged recombinant ULK2 or Kapβ2 preparations was subsequently added to separate samples followed by 2 hour incubation at 4°C with end-over-end rotation to allow for association between ULK2 and Kapβ2. Associated protein complexes were collected using the slurry of glutathione agarose beads, and washed extensively. After resuspension in 2x Laemmli sample buffer, samples were analyzed on a 10% SDS-PAGE gel, and western blotting was performed using Kapβ2 or ULK2 antibodies.

### Immunoblotting

Pulled-down or immunoprecipitated ULK2 (or Kapβ2) was resolved on 10% SDS-PAGE gels and transferred to a nitrocellulose membrane. Membranes were incubated in blocking buffer (5% dried skimmed milk in PBS and 0.05% Tween-20), and subsequently probed with specific antibodies, followed by a horseradish peroxidase-conjugated secondary antibody. Immune complexes were detected using a commercial western blotting detection system (Pierce, Rockford, IL, USA).

### Confocal microscopy

HEK293 cells were seeded overnight at 60% confluence onto culture slides coated with human fibronectin (SPL, Korea). The following day, cells were transfected with the ULK2/EGFP construct, and allowed to grow for an additional 48 hours. Cells were washed several times with ice-cold PBS and fixed in 2% paraformaldehyde for 10 minutes. Fixed cells were permeabilized with 0.1% Triton X-100 for 10 minutes and blocked for 2 hours in PBS containing 5% BSA (Aurion, The Netherlands) and 0.1% Tween. Following incubation with a polyclonal (rabbit) or monoclonal (mouse) antibody against Kapβ2(1:100), ULK2 (1:100), LC3 II (1:100) or WIPI (1:100) (in 5% BSA-PBS; Bio-Protocol, Palo Alto, CA, USA) overnight at 4°C, the slides were washed three times with 0.01% PBS-Tween. Alexa Fluor 568 or 488-conjugated donkey anti-rabbit (1:200) or anti-mouse (1:200) (in 5% BSA-PBS; Molecular Probes, Inc., Eugene, OR, USA) was used as a secondary antibody. Confocal microscopy analysis was performed using an LSM710 (Zeiss, Germany) at the Center for Research Instruments and Experimental Facilities of Chungbuk National University. Using Profile in the ZEN program which was provided by the manufacturer, the co-localizations of proteins were observed and confocal microscopic images scanned. The nuclear or cytoplasmic fluorescence intensity profile of ULK1/2 was evaluated from the fluorescence images, and the nuclear-to-total cell fluorescence (Fn/t) ratio was obtained using the ZEN program [13,17,18]. Pearson's correlation coefficient (PCC) of the co-localization between ULK2 and Kapβ2 was measured with an LSM710 (Zeiss, Germany).

### FACS analysis

Cells were transfected with ULK2 (WT), PY mutant (P242A or P794A), or EGFP vector, and the rate of apoptosis was measured using the Annexin V-PE apoptosis detection kit (BD Biosciences, NJ, USA), according to the manufacturer’s instructions. Cells were vortexed gently, incubated for 15 minutes at 25°C in the dark, and 400μl binding buffer added to each tube. Within 1 hour, fluorescence-activated cell sorting (FACS) was performed using a FACS Calibur (BD Sciences) at the Core Facility of Chungbuk National University.

### Autophagy assay

To measure autophagy, we used the LC3 western blotting method (Bio-Protocol.org) following the manufacturer’s guide, in the same manner as described in the “Immunoblotting” section above using an antibody against LC3. Each assay was performed 5 times.

### Statistical analysis

Numerical results were described as means ±SD from at least four independent experiments performed in triplicate. Differences in the measured variables between control and experimental groups were analyzed using a Student’s t-test. A statistically significant difference was accepted at * P < 0.05 or ** P < 0.01.

## Results

### Endogenous ULK2 interacts with karyopherin β2 (Kapβ2) and localizes in the nucleus

**Fig 1:**
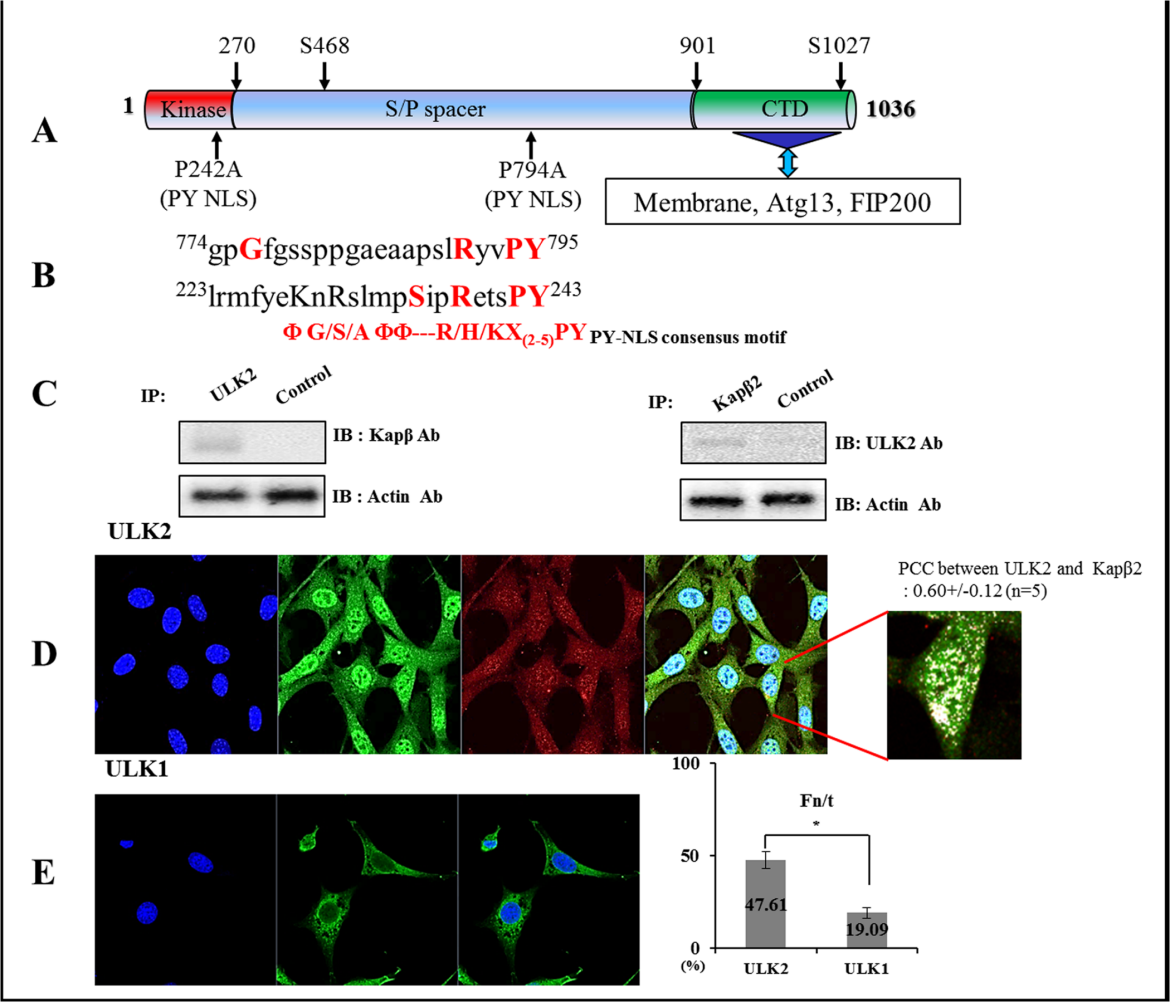
Interaction between the putative PY-NLS motif of ULK2 and Kapβ2 and the subcellular localization of ULK2 and Kapβ2. (A) ULK2 (Gene ID; KIAA0623) contains two putative-conserved Kapβ2 binding motifs. (^220^qdlrmfyeKnRslmpSipRetsPY^243^) in its protein kinase domain and (^774^gpGfgssppgaeaapslRyvPY^795^) within the serine/proline (S/P)-rich space domain. Two point mutations (P242A and P794A) were prepared to define the binding motif. Mutated sequences are indicated with arrows. P794A mutant (^774^gpGfgssppgaeaapslRyvPY^795^ changed to ^774^gpGfgssppgaeaapslRyvAY^795^) or P242A mutant (^220^qdlrmfyeKnRnotslmpSipRetsPY^243^ changed to ^220^qdlrmfyekKnRslmpSipRetsAY^243^) were constructed. Putative PKA-phosphorylation sites (Ser468 and Ser1027) are also indicated with arrows [1,9]. Regions of the protein in the C-terminal domain (CTD) that are involved in membrane attachment and interaction with Atg13-focal adhesion kinase family-interacting protein 200 (FIP200) are indicated [9,28]. (B) Two putative ULK2 PY NLS motifs were aligned with the defined PY NLS motif. Both motifs (^774^gpGfgssppgaeaapslRyvPY^795^ and ^220^qdlrmfyeKnRslmpSipRetsPY^243^) matched the consensus PY-NLS motif of (ΦA/G/SΦΦ—R/K/H)*X*_2–5_PY well [1,11,14–16]. (C) Following immunoprecipitation (IP) with an anti-ULK2 antibody, immunoblotting (IB) was performed using an antibody against Kapβ2 (left). Conversely, anti-Kapβ2 immunoprecipitated complexes were subjected to immunoblotting using an anti-ULK2 antibody (right). Co-immunoprecipitation of Kapβ2 with ULK2 confirms the presence of a ULK2-Kapβ2 complex in the cell. As a control for immunoprecipitation, an unrelated antibody against EGFP was used. For the control of immunoblotting, an antibody against actin was used (bottom). (D) Confocal fluorescence micrographs showing endogenous ULK2 and Kapβ2 in HEK293 cells. These proteins were visualized by immunofluorescence in fixed and permeabilized cells using monoclonal or polyclonal antibodies against human Kapβ2 or ULK2, and Alexa Fluor 568-conjugated donkey anti-rabbit IgG or Alexa Fluor 488-conjugated mouse anti-rabbit IgG. The yellow pattern resulting from the merging of red and green colors indicates co-localization of the proteins in the cytoplasm (white color in the nucleus). The cell’s nuclear region was visualized using Hoescht staining (blue color). To determine the co-localization of ULK2 and Kapβ2, the enlarged co-localization image of the specific merged region is shown (white color). PCC between ULK2 and Kapβ2 was measured by quantitative confocal microscopy. (E) Confocal fluorescence micrographs showing localization of endogenous ULK1 (green color; not containing a PY-NLS motif), which is in the cytosol but not in the nucleus in HEK293 cells, for comparison. The cell’s nuclear region was visualized using Hoescht staining (blue color). The image of Kapβ2 is not present. Fluorescence images were analyzed to calculate the nuclear-to-total fluorescence ratio (Fn/t; see Materials and methods). The graph shows the percentage of ULK1 and ULK2 localization in the nucleus; the mean ± SEM (error bars; n ≥ 10) from a single assay representative of three separate experiments (* P<0.05; ** P<0.01; Student’s t-test is used throughout).

Most Kapβ2-associated proteins have Kapβ2 binding motifs (R/H/KX_(2–5)_PY) [11,14–16]. We found that ULK2 contained two potential Kapβ2 binding motifs; one (^220^qdlrmfyeKnRslmp**s**ipRetsPY^243^) within its kinase domain, and a second motif (^774^gpgfgssppGaeaapslRyvPY^795^) within its S/P space domain (Fig 1A). The presence of two putative conserved Kapβ2 binding motifs in ULK2 suggested that ULK2 is able to bind to Kapβ2 (Fig 1B).

**Fig 2:**
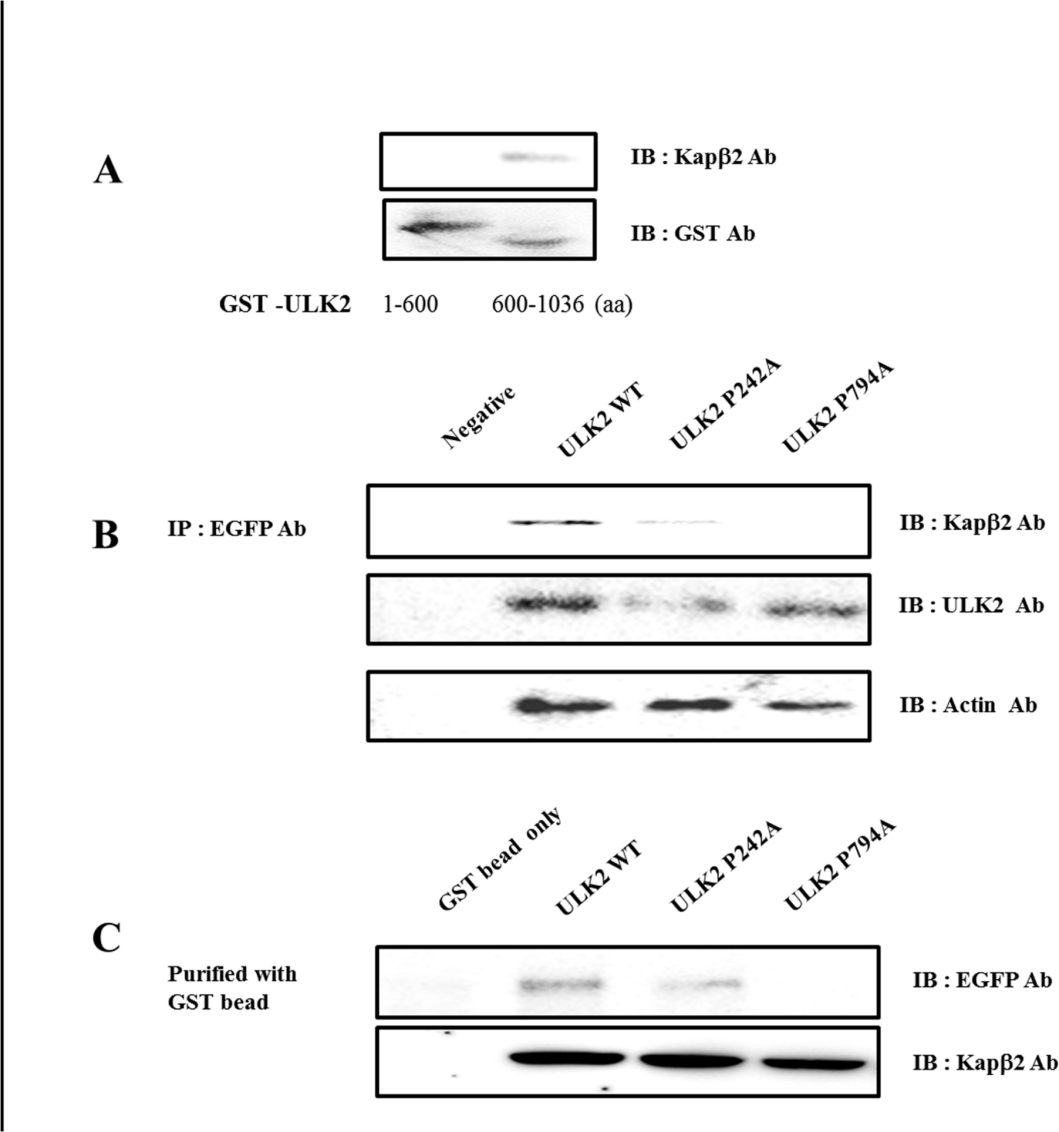
^774^gpgfgssppGaeaapslRyvAY^795^ in ULK2 is required for the interaction between ULK2 and Kapβ2. (**A**) Pull-down analysis of Kapβ2 with GST fusion C-terminal ULK2 1–600 or 601–1036 mutants. GST-fusion proteins encompassing the C-terminal of ULK2 were constructed and expressed in *E*. *coli*. Approximately 0.1mg of 1–600 or 601–1036 fusion proteins, bound to glutathione-sepharose beads (bottom lane), were incubated with HEK293 cell lysates. (**B**) Co-immunoprecipitation of ULK2 WT, P794A, or P242A with Kapβ2. HEK293 cells were transiently transfected with the EGFP-ULK2 WT or P794A plasmid. After 48 hours, the cells were lysed, and western blotting carried out with an anti-EGFP antibody and protein A agarose beads. Western blotting assays were performed with rabbit anti-ULK2 or mouse anti-Kapβ2 antibodies. The large size (~110kDa) of Kapβ2 is shown here. To monitor the amount of total protein in the cell lysate, western blotting was also performed with an anti-actin antibody. The negative control was untransfected HEK293 cell lysate. (C) Interaction between ULK2 and WT, P794A, or P242A *in vitro*. HEK293 cells were transiently transfected with the EGFP-ULK2 WT, P794A, or 242A plasmid. After 48 hours, the cells were lysed, and pull-down assays were conducted with GST-Kapβ2 beads. Western blotting was performed with rabbit anti-ULK2 or mouse anti-Kapβ2 antibodies. The large size (~110 kDa) of Kapβ2 is shown. GST-beads were used as the negative control.

Due to the fact that ULK2 contains two putative Kapβ2 binding motifs (Fig 1B), our aim was to determine whether endogenous Kapβ2 forms a protein complex with ULK2 in HEK293 cells. As shown in the left lane of Fig 1C, the ULK2 immunoprecipitate contained Kapβ2. Antibodies directed against Kapβ2 were also able to successfully capture ULK2 from the same lysates, corroborating the hypothesis that the two proteins physically associate (Fig 1C, right lane). In the pull-down experiment with the pGEX-5X-1 Kapβ2 fusion protein (the construct was kindly provided by Dr. Chook) and HEK293 cell lysate, the capture of ULK2 by Kapβ2 was also observed (Fig 2C). Furthermore, we examined whether ULK2 is associated with Kapβ2 in cells using confocal microscopy. Endogenous ULK2 (green) and Kapβ2 (red) were indeed co-localized in both the nucleus and cytoplasm (yellow) (Fig 1D).

Fig 1D emphasizes the co-localization of ULK2 and Kapβ2 (white spot) in the enlarged image of the specific merged region. Pearson's correlation coefficient (PCC) of the co-localization between ULK2 and Kapβ2 was determined using an LSM710 [17,18]. The PCC value (0.60+/− 0.12; n = 5) indicates that the coexistence of ULK2 and Kapβ2 in HEK293 cells positively occurs with roughly a 60% probability. In other cell lines, namely 3T3, MDCK2, and HepG2, endogenous ULK2 was found predominantly in the nucleus, not in the cytosol (data not shown).

Confocal observations of ULK1 were also performed for comparison with ULK2 [1], because ULK1 does not contain a Kapβ2 binding motif [1,14]. In contrast to the ULK2 results (Fig 1D), endogenous ULK1 was observed mainly in the cytosol, not in the nucleus (Fig 1E). In contrast to previous studies [2–4], we found dominant localization of endogenous ULK2 in the nucleus, not the cytoplasm (Fig 1D).

The nuclear-to-cytoplasmic fluorescence ratio of ULK1 or ULK2 was also measured using the ZEN program [17,18]. The Fn/t ratio of ULK2 (47. 61+/−4.59; n = 10) was 2.4 times higher than that of ULK1 (19.09+/−2.87; n = 10) (Fig 1E, graph). The results indicate that even though ULK2 is present evenly in both the nucleus and the cytoplasm (Fig 1D), ULK1 is dominantly found in the cytoplasm (Fig 1E). Thus, our observations strongly suggest that approximately 60% of endogenous ULK2 interacts with Kapβ2 in HEK293 cells, and that roughly 47% of ULK2 is localized in the nucleus from the cytoplasm, in contrast to ULK1, which is detected mainly in the cytoplasm (Fig 1E, histogram).

### The (^774^gpgfgssppGaeaapslRyvPY^795^) motif of ULK2 is a functional PY-NLS motif that can bind to Kapβ2

As shown in Fig 1C and 1D, ULK2 interacts with Kapβ2 *in vitro*. Due to the fact that ULK2 contains two well-conserved Kapβ2 binding motifs, we set out to determine which of the two ULK2 motifs binds to Kapβ2 in HEK293 cells. At first, we assumed that the (^774^gpgfgssppGaeaapslRyvPY^795^) motif of ULK2 was the true PY-NLS motif [1,14]. In order to test this, we constructed GST-ULK2 WT (1–600 aa) or (601–1036 aa); these constructs contain the potential Kapβ2 binding motif (^220^qdlrmfyeKnRslmpsipRetsPY^243^) in the kinase domain, or the potential Kapβ2 binding motif (^774^gpGfgssppgaeaapslRyvPY^795^) within the S/P space domain, respectively. Approximately 1mg of 1–600, or 601–1036 fusion protein bound to glutathione-sepharose beads was incubated with HEK293 cell lysates. Pull-down analysis of Kapβ2 with GST fusion N-terminal ULK2 1–600 or C-terminal 601–1036 proteins expressed in *E*. *coli*, confirmed that the (^774^gpGfgssppgaeaapslRyvPY^795^) motif within the S/P space domain of ULK2 is the primary PY-NLS motif of ULK2, because it pulled down Kapβ2 (Fig 2A). Both (^220^qdlrmfyeKnRslmpSipRetsPY^243^) and (^774^gpgfgssppGaeaapslRyvPY^795^) motifs are found only in ULK2, and not in ULK1, suggesting that ULK2 and ULK1 potentially have different functions [1,14].

In order to demonstrate the interaction between ULK2 and Kapβ2 through its putative PY-NLS motif, a co-immunoprecipitation experiment was conducted (Fig 2B). EGFP-ULK2 WT and the point mutations (P242A or P794A) were transfected separately into HEK293 cells. After 48 hours, the cells were lysed, and the immunoprecipitation was conducted with a rabbit anti-EGFP antibody. To examine whether the immunoprecipitant brought down Kapβ2 together with ULK2, western blotting was performed with mouse anti-Kapβ2, anti-ULK2 or mouse anti-actin antibodies (1:2000 dilution). As shown in Fig 2B, EGFP-ULK2 WT and the P242A mutant was able to specifically bring down the endogenous Kapβ, while the P794A mutant failed to interact with Kapβ2. Thus, these results suggest that the putative PY-NLS motif present in the 774–795aa fragment of ULK2 is responsible for the binding to Kapβ.

To gain more evidence for an interaction between ULK2 and Kapβ2 through its putative PY-NLS motif, the pull-down experiment with the GST-Kapβ2 protein purified in *E*. *coli* was conducted (Fig 2C). The EGFP-ULK2 WT or EGFP-ULK2 PY-NLS mutant (P242A or P794A) was transfected into HEK293 cells. After 48 hours, the cells were lysed, and pull-down of the cell lysate was conducted with GST-Kapβ2 beads. Western blotting assays were performed with rabbit anti-EGFP or mouse anti-Kapβ2 antibodies. As shown in Fig 2C, GST-Kapβ2 was able to specifically pull down EGFP-ULK2 WT and the P242A mutant, while this fusion protein failed to interact with the P794A mutant. Similar results were also seen in Fig 2B, indicating that this motif present in the 774–795aa fragment is crucial for the interaction of ULK2 with Kapβ2 (Fig 2C). Therefore, these results demonstrate that the PY-NLS motif (aa774–795) in the ULK2 S/P space domain is the main functional PY-NLS motif in ULK2 (Fig 1A).

### Interaction between exogenous ULK2 and Kapβ2 is required for nuclear localization of ULK2 in HEK293 cells

**Fig 3:**
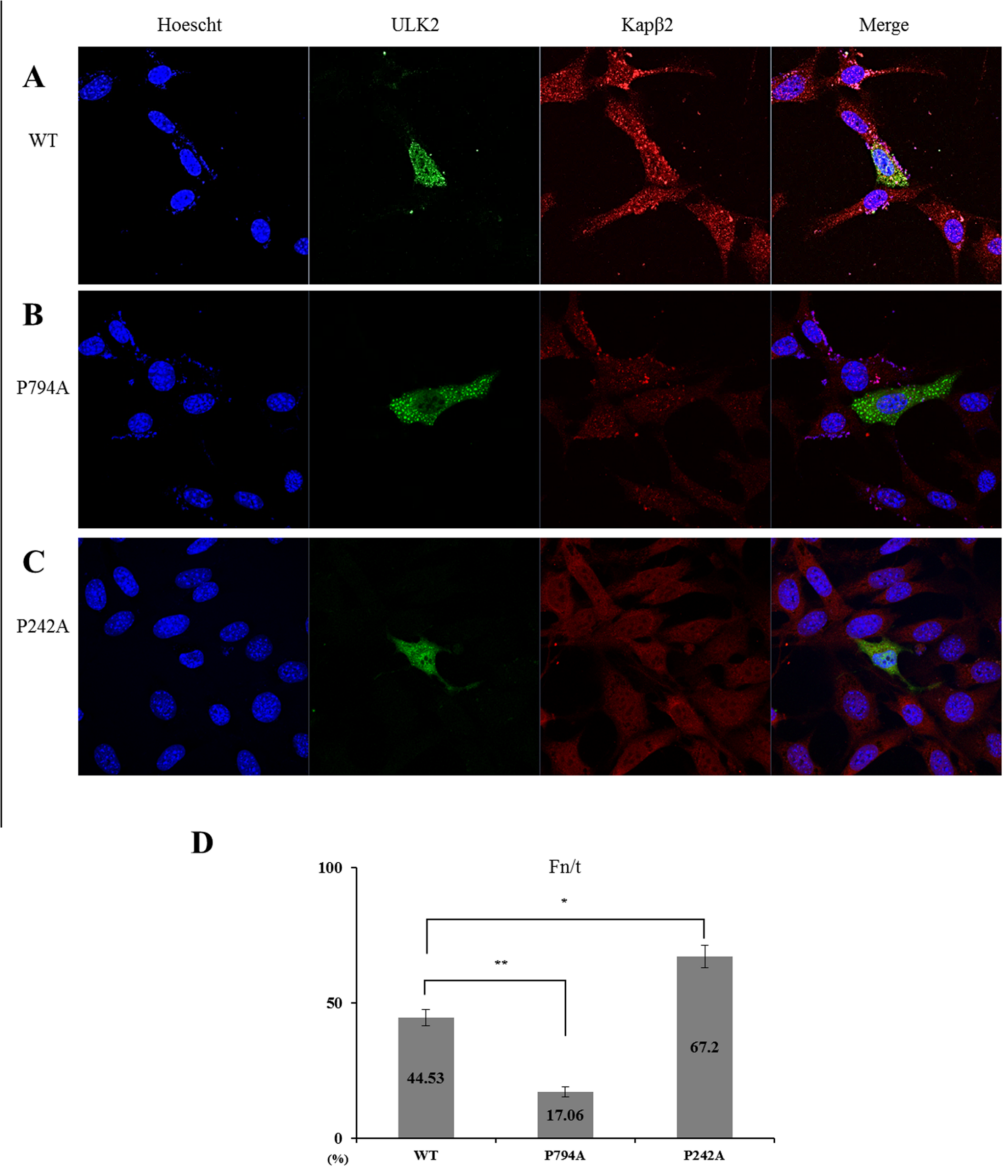
Subcellular localization of exogenous ULK2 PY mutants. A) Confocal fluorescence micrographs of EGFP-ULK2 WT, P794A, and P242A in HEK293 cells. All EGFP constructs fluoresced green, while Kapβ2 fluoresced red. Transfected EGFP-ULK2 WT merged with Kapβ2 is predominantly in the nucleus (**A**). Yellow color resulting from merging of the red and green indicates co-localization of both proteins. This is found mainly in the nucleus, similar to the results obtained for endogenous ULK2 shown in Fig 1C. The transfected EGFP-ULK2 PY-NLS mutant (P794A, which is not detected in the nucleus) is not co-localized with Kapβ2 in the cytoplasm either (**B**). A greater number of puncta are shown (**B**). Subcellular localization of the ULK2 P242A mutant is also visualized in (**C**) This mutant is localized with Kapβ2 mainly in the nucleus (yellow color). The cell’s nuclear region is visualized with Hoescht staining (blue). Representative images based on five replicate experiments for each construct are shown. Fluorescence images of **(A-C)** were analyzed to calculate the nuclear-to-total fluorescence ratio (Fn/t). Histograms indicate SD (error bars; n ≥ 10) from a single assay of three separate experiments (* P < 0.05; ** P < 0.01). The graph on the **Fig 3D** shows the mean ± SEM (error bars; n ≥ 10) from a single assay representative of three separate experiments (* P < 0.05; ** P < 0.01).

To better understand the effects of the interactions between ULK2 and Kapβ2, confocal microscopy was performed (Fig 3). We investigated whether the putative PY-NLS sequence (aa774–795) in ULK2 could direct the import of this protein to the nucleus, from the cytoplasm.

In a finding consistent with the endogenous ULK2 results shown in Fig 1D, exogenous EGFP-ULK2 WT (green) and Kapβ2 (red) were localized together in the plasma membrane (yellow) (Fig 3A). However, the exogenous EGFP-ULK2 PY-NLS P794A mutant (green) was not co-localized with Kapβ2 (red) (Fig 3B), likely due to the mutation in its Kapx2 binding site. The EGFP-ULK2 PY-NLS P794A mutant (green) was localized to the cytoplasmic region, rather than to the nucleus (Fig 3B). However, the exogenous EGFP-ULK2 P242A mutant (green) was co-localized with Kapβ2 (red) in the nucleus (Fig 3C), likely due to its Kapβ2 binding site. For the quantification of ULK2 WT, PY-NLS P794A mutant, or P242 mutant nuclear localization, each Fn/t ratio was determined. As shown in the histogram in Fig 3D, the ULK2 WT (44. 53+/−3.04; n = 10) and the P242A mutant (67. 2+/−4.19; n = 10) was 2–3 times higher than that of ULK2 PY-NLS (P794A) mutant (17.06+/−1.87; n = 10). These results indicate that the P794A mutation ablates its nuclear localization. Therefore, we have called it a PY-NLS mutant. Taken together, these data strongly suggest that Kapβ2 interacts with ULK2 through the latter protein’s PY-NLS consensus motifs (^774^gpgfgssppGaeaapslRyvPY^795^) in the S/P space domain (Fig 1A), and that protein-protein interaction between ULK2 and Kapβ2 is required for nuclear localization of ULK2 (Fig 3).

### Cytoplasmic localization of ULK2 enhances its autophagic activity

**Fig 4:**
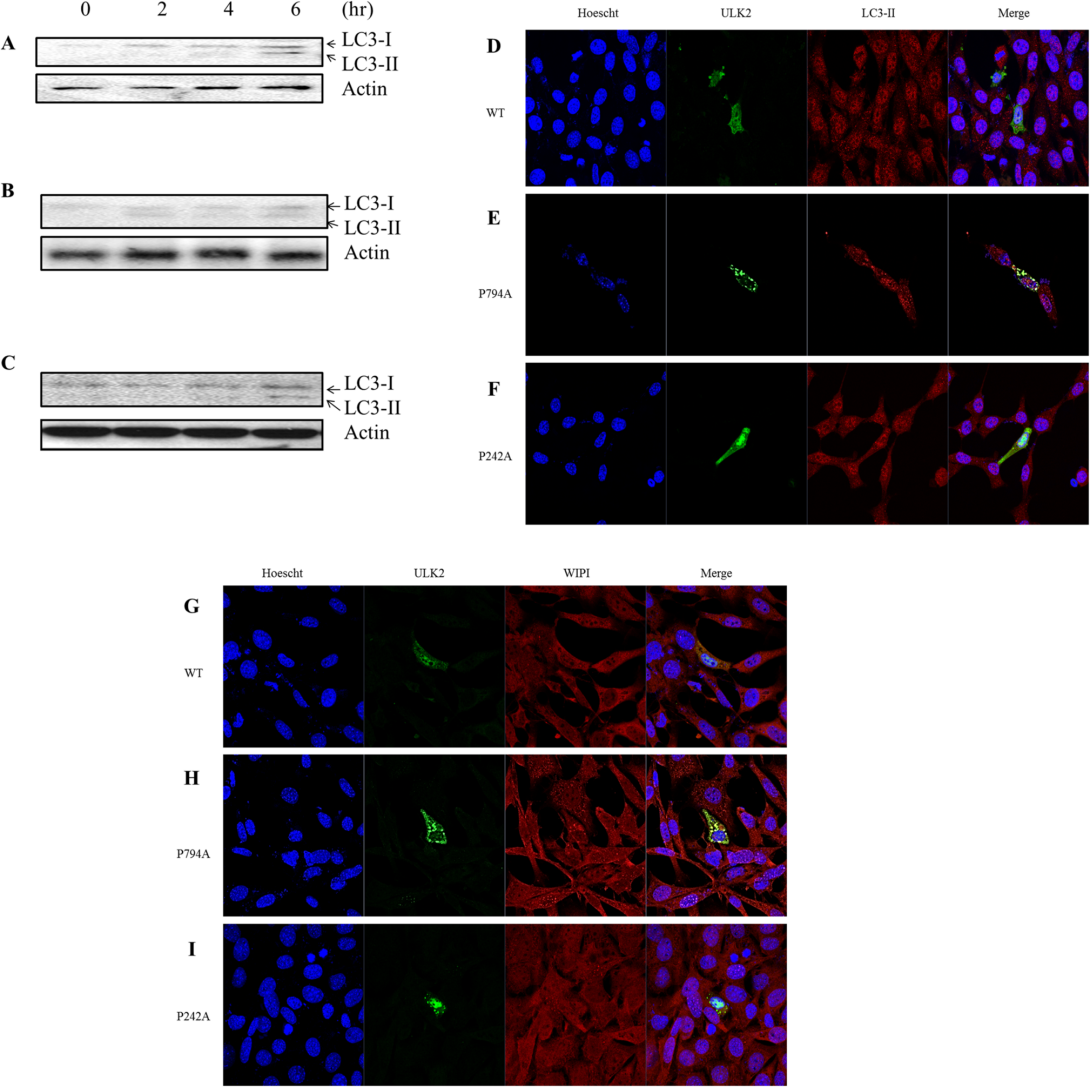
Autophagic ability of WT and mutant ULK2. EGFP ULK2 WT or its mutants (P242A, P794A) were transfected in HEK293 cells as described in the Materials and Methods section. Accumulation of microtubule-associated protein light chain 3-II (LC3-II) in cells is indicated with arrows. The same number of HEK293 cells were transfected with ULK2 WT (**A**) or its mutants P794A (**B**), P242A (**C**) for 48 hours, then LC3-II appearance was analyzed by western blotting at different time points (0, 2, 4, 6 hours) after starvation induction. The amount of ULK2 protein was monitored using an ULK2 antibody (lower lane). To visualize co-localization of ULK2 WT (**D**), P794A (**E**), and P242A (**F**) with endogenous LC3-II in HEK293 cells, cells were stained with an LC3-II antibody 12 hours after starvation induction. Confocal fluorescence micrographs were taken in a similar manner to that described in the legend to Fig 3. To visualize co-localization of ULK2 WT (**G**), P794A (**H**), and P242A (**I**) with endogenous human WD-repeat protein interacting with phosphoinositides (WIPI; another autophagic marker protein) in HEK293 cells, cells were stained with a WIPI antibody 12 hours after starvation induction. Confocal fluorescence micrographs (**G-I**) were taken in a similar manner to that described in the legend to D-F. The antibody against LC3-II or WIPI was used according to the manufacturer’s recommendations (see Materials and Methods section).

To evaluate the effect of the ULK2 PY-NLS mutant (P794A) on autophagy, we assayed the total amount of microtubule-associated protein light chain 3 (LC3), which is the mammalian homologue of the autophagy-related Atg8 in yeast, as described in the Materials and Methods section [19,20]. The total amount of LC3 was used as a marker of autophagy. HEK293 cells, incubated in glucose-free medium, were transfected with each EGFP-ULK2 (WT, P242A, and the PY-NLS mutant) expression vector. This was followed by immunoprecipitation using an EGFP monoclonal antibody (Fig 4). ULK2 proteins were chased for the indicated time periods (0, 2, 4, 6 hours after starvation induction), and subsequently subjected to SDS-PAGE, followed by western blotting analysis with a polyclonal anti-LC3 antibody [19,20]. As a control for the amount of total cellular protein, the level of actin was monitored in each sample with an anti-actin antibody (Fig 4A–4C). We used the P242A mutant, which was transported into the nuclear region, as the negative control (Fig 4C).

As shown in Fig 4B, LC3-II (a marker for the appearance of autophagosomes) was detected in cells transfected with the ULK2 PY-NLS mutant 2 hours earlier than in the cells transfected with ULK2 WT (Fig 4A) or the P242A mutant (Fig 4C), suggesting that the cytoplasmic ULK2 PY-NLS mutant facilitates faster autophagosome (or puncta) appearance than nuclear-localized ULK2. Further, we performed confocal microscopy of endogenous LC3 and each ULK2 construct (Fig 4D–4F) 12 hours after starvation induction. Consistent with Fig 4B, LC3-II in cells transfected with the ULK2 PY-NLS mutant (Fig 4E) co-localized with the mutant protein in the cytoplasm compared with LC3-II in cells transfected with the ULK2 WT (Fig 4D) or the P242A mutant (Fig 4F), and these proteins were predominantly found in the nucleus.

**Fig 5:**
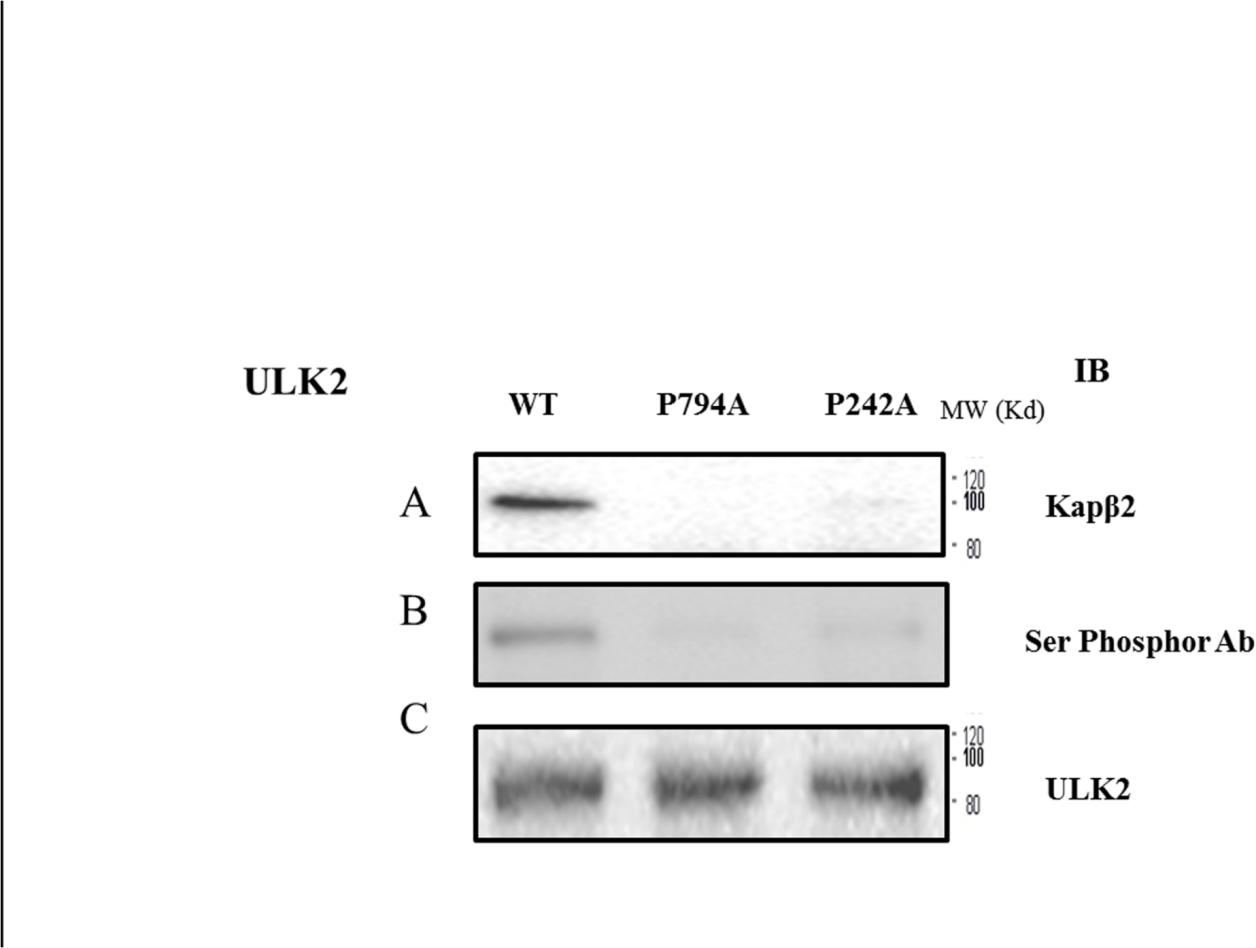
Comparison of the serine phosphorylation status of ULK2 WT and its mutants. EGFP ULK2 WT and its mutants (P242A and P794A) were purified with an EGFP antibody, as described in the Materials and Methods section. Phosphorylation of ULK2 was detected with IB using anti-phosphor Ser (upper lane) or ULK2 antibodies. The amount of ULK2 protein in the experiment was monitored using a ULK2 antibody (lower lane) [1,3,5].

For additional supporting evidence, we conducted confocal microscopy of an endogenous human WD-repeat protein interacting with phosphoinositides (WIPI, another autophagy marker protein) [8,9] and each ULK2 construct (Fig 4G–4I) 12 hours after starvation induction. WIPI in cells transfected with the ULK2 PY-NLS mutant (Fig 5H) also dominantly co-localized with the mutant protein in the cytoplasm, compared with WIPI in cells transfected with ULK2 WT (Fig 4G) or the P242A mutant (Fig 4I). Similar to Figs 1D, 3A and 4D, the ULK2 WT protein is also predominantly found in the nucleus (Fig 4G). Overall, the subcellular localization of each ULK2 mutant in Fig 4G–4I (with the WIPI antibody) was similar to the result seen in Fig 4D–4F (with LC3 Ab).

Thus, the confocal microscopy with the WIPI antibody also confirmed that the cytoplasmic ULK2 PY-NLS mutant facilitates faster autophagosome (or puncta) appearance than does the nuclear-localized ULK2 (Fig 4H). These results are also consistent with the results shown in Fig 3B, there are a greater number of puncta (assumed to be autophagosomes) in the confocal image compared with that shown in Fig 3A. To confirm the confocal observation, a co-immunopreciptation experiment was performed and a strong protein complex formation of the ULK2 PY-NLS mutant with WIPI was observed, compared with that of the WT or P242A mutant (data not shown). Taken together, these data support the notion that ULK2 cytoplasm localization by the PY-NLS mutation (P724A) enhances its autophagic activity. Furthermore, the subcellular localization of each ULK2 construct (Fig 4D–4F or Fig 4G–4I) confirms again that the ^774^gpGfgssppgaeaapslRyvPY^795^ motif of ULK2 is its NLS motif.

### Localization of ULK2 changes the phosphorylation of its serine residues

As shown in Figs 1 and 2, the S/P space domain of ULK2 contributes to its physical interaction with Kapβ2. In order to characterize the functional consequences of this protein-protein interaction in the nucleus, we transiently expressed EGFP-ULK2 WT, P242A, and P794A mutant proteins in HEK293 cells, and compared their serine phosphorylation status [3]. Phosphorylation of several serine residues in ULK2, including serine-phosphorylation of Ser335 (corresponding to Ser341 in ULK1), have been reported [1,3]. Based on the assumption that less phosphorylated ULK2 forms a protein complex in the cytoplasm, we examined whether ULK2 subcellular localization affects its serine residue phosphorylation status. An anti-ULK2 antibody was used to monitor ULK2 expression (Fig 5A). In the same samples, an anti-Kapβ2 antibody was used to determine whether Kapβ2 co-immunoprecipitated with exogenous ULK2. Consistent with the results shown in Fig 2D, we observed Kapβ2 in cells transfected with the EGFP-ULK2 WT and the EGFP-ULK2 P242A mutant immunocomplexes, but not in the immunocomplex from EGFP-ULK2 PY-NLS mutant (P794A) (Fig 5A), which proves once again that ULK2 interacts with Kapβ2 through its NLS consensus motif (^774^gpGfssppgeaapslRyvPY^795^) in the S/P space domain (Fig 5A).

As shown in Fig 5B, western blotting using an anti-phosphor Ser antibody with the immunoprecipitant obtained with an anti-EGFP antibody revealed that serine phosphorylation of EGFP-ULK2 PY-NLS is about 15% of that of EGFP-ULK2, even though the same amount of EGFP-ULK2 and EGFP-ULK2 PY-NLS mutant protein was investigated (Fig 5C). Thus, the results suggest that cytoplasmic-localized ULK2 is less serine phosphorylated than nuclear-localized ULK2, even though the specific serine phosphorylation sites and the corresponding protein kinases were not determined here.

### Phosphorylation of ULK2 Ser1027 residue exerts its dissociation with Atg13 and FIP200, nuclear localization, and autophagy

Next, in order to determine which ULK2 specific phosphorylation site is related to the control of its subcellular localization, we at first assumed that it would be the ULK2 Ser1027 residue (^1024^RRlSA^1028^) in its C-terminal domain (CTD), where there is overlap with its Atg13 and FIP200 association site (Fig 1A). Furthermore, because the putative site in ULK2 is well matched with the consensus protein kinase A (PKA) substrate information [R-R-X-(S/T)-Φ], PKA may phosphorylate the 1027 residue of ULK2, as one of its specific substrate proteins [21,22]. Preliminarily, we observed that PKA phosphorylates the Ser1027 (not the Ser468) residue of ULK2 with the PKA substrate phosphorylation specific antibody (Fig 1A, our unpublished data). Thus, we hypothesized that PKA-mediated phosphorylation of the ULK2 Ser1027 residue exerts its functional dissociation with Atg13 and FIP200, nuclear localization, and autophagy [1–9]. It has been previously reported that the subcellular localizations of several proteins are also regulated through PKA phosphorylation [23–26].

**Fig 6:**
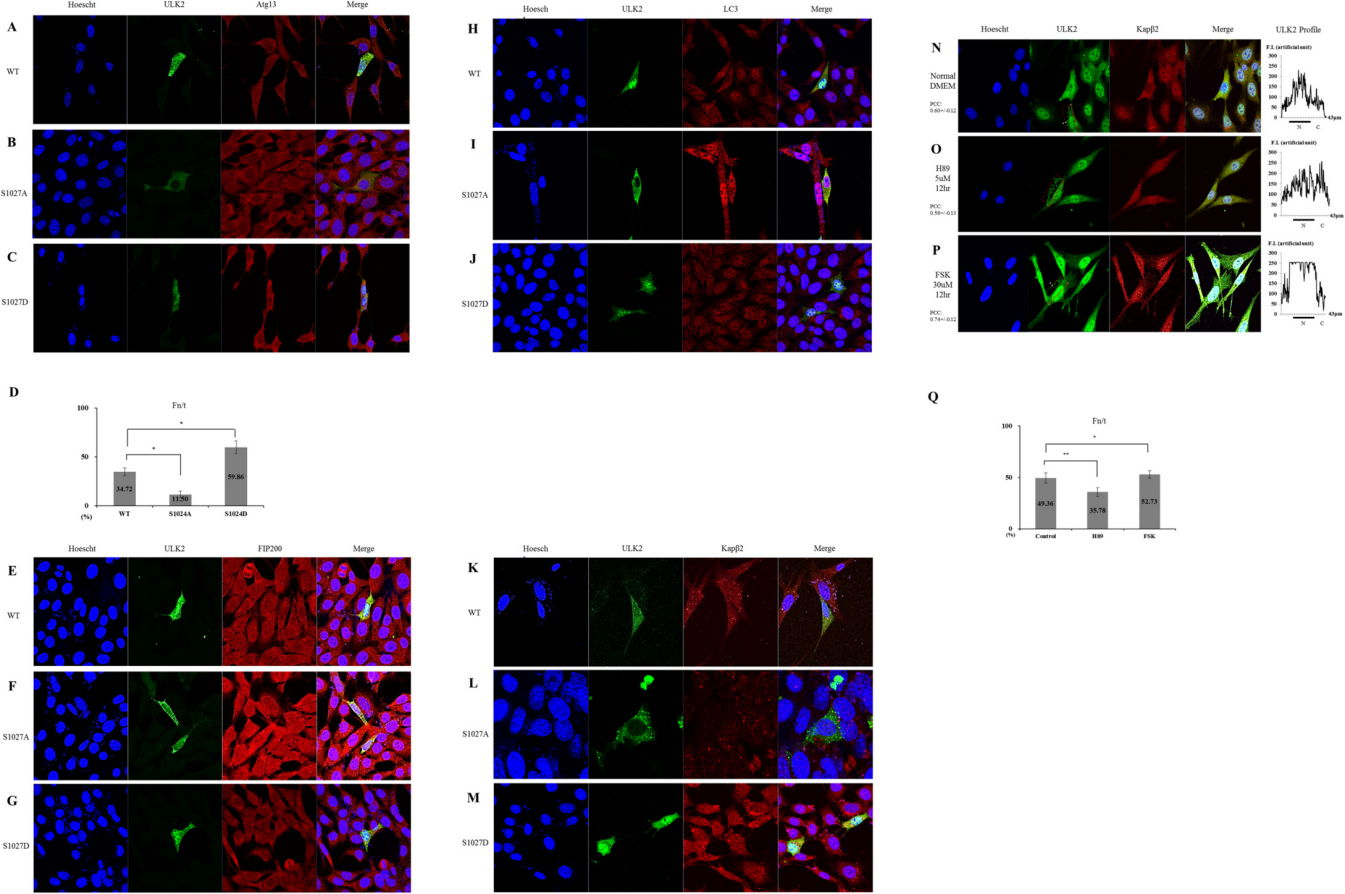
Subcellular localization and autophagic ability of ULK2 Ser1027 mutants. To visualize co-localization of EGFP-ULK2 WT, S1027A, and S1027D (green) with endogenous human Atg13 **(A-C)** or FIP200 **(E-G)** in HEK293 cells, cells were stained with an Atg13 or FIP200 antibody (red color) 12 hours after starvation induction. The antibodies against Atg13 and FIP200 were used according to the manufacturer’s recommendations (see Materials and Methods section). To compare co-localization of ULK2 WT, S1027A, and S1027D with endogenous LC3-II (as a marker of autophagy) in HEK293 cells, cells were stained with an LC3-II antibody (red color) 12 hours after starvation induction **(H-J)**. To further examine co-localization of ULK2 WT, S1027A, and S1027D with endogenous Kapβ2 in HEK293 cells, cells were stained with a Kapβ2 antibody (red color) 12 hours after starvation induction **(K-M)**. Fluorescence images of Fig **6A–6C** were analyzed to calculate the nuclear-to-total fluorescence ratio (Fn/t). Histograms show the mean of three experiments, bars indicate SD (error bars; n ≥ 10) from a single assay of three separate experiments (* P < 0.05). The graph on the **Fig 6D** shows the percentage of ULK2 WT, S1027A, or S1027D mutant localization in the nucleus. The endogenous ULK2 in HEK293 cells was visualized with a PKA inhibitor (H89 5μM for 12 hours) and activator (FSK 30μM for 12 hours). ULK2 was stained green and kapβ2 was stained red. The cell’s nuclear region was visualized with Hoescht staining (blue color) **(N-P)**. Confocal fluorescence micrographs were taken in a similar manner to that described in the legend to Fig 3. PCC between ULK2 and Kapβ2 was measured using quantitative confocal microscopy (n = 5). Each PCC is indicated on the left side of **Fig 6N–6P**. The nuclear (N) or cytoplasmic (C) fluorescence intensity (FI) profile (each 43μm distance) of ULK2 is shown on the right side of **Fig 6N–6P**. The bar indicates the nuclear region. Fluorescence images of **Fig 6N–6P** were analyzed to calculate the nuclear-to-total fluorescence ratio (Fn/t). Histograms indicate SD (error bars; n ≥ 10) from a single assay of three separate experiments (* P < 0.05; ** P < 0.01). The graph on the **Fig 6Q** shows the percentage of endogenous ULK2 localization in the nucleus, depending on H89 or FSK treatment.

In order to investigate our speculation, we constructed EGFP-ULK2 WT, S1027A (dephosphorylated analogous mutant), and S1027D (phosphorylated analogous mutant) using site-directed mutagenesis, and their subcellular localization and protein-protein interaction with Atg13 (Fig 6A–6C), FIP200 (Fig 6E–6G), and LC3-II (Fig 6H–6J) were compared. To further analyze the binding capacity between Kapβ2 and each ULK2 protein, WT, S1027A, or S1027D, confocal microscopy was also conducted (Fig 6K–6M).

As expected, ULK2 S1027A was not transported into the nucleus (Fig 6B), as compared with ULK2 WT or S1027D (Fig 6A or 6C). Using quantitative confocal microscopy, the Fn/t ratio of the WT (34.72+/−4.01; n = 10), S1027D (59.86+/−6.58; n = 10), and S1027A (11. 50+/−3.48; n = 10) was also determined (Fig 6D, histogram). The results suggest that the PKA phosphorylated analogous mutant (S1027D) is strongly transported to the nucleus, whereas this transport is not seen with the PKA dephosphorylated analogous mutant (S1027A), which remains in the cytoplasm. Therefore, it seems to be clear that the phosphorylation of the ULK2 Ser1027 residue by PKA promotes ULK2 nuclear localization, while the dephosphorylated protein remains in the cytoplasm.

Furthermore, ULK2 S1027A interacted with Atg13 (Fig 6B) and with FIP200 (Fig 6F) in the cytoplasm (not in the nucleus). In contrast, ULK2 WT and S1027D were mainly transported to the nucleus, but interacted much less with Atg13 (Fig 6A and 6C) or with FIP200 (Fig 6E and 6G), suggesting that the phosphorylation on the Ser1027 residue by PKA is one of the major regulatory events for ULK2 subcellular localization, similar to other PKA substrate proteins [23–26].

In order to compare the autophagic activity of ULK2 WT, S1027A, and S1027D, the endogenous LC3-II (as marker of autophagy) in HEK293 cells expressing these proteins was stained with the LC3-II antibody 12 hours after starvation induction (Fig 6H–6J). ULK2 S1027A (which was not transported to the nucleus) was well co-localized with LC3-II (more yellow specks than with WT and S1027D) in the cytoplasm (Fig 6I), while WT and S1027D (both were transported to the nucleus) were less well-merged (Fig 6H and 6J). Furthermore, the number of puncta seen (presumably autophagosome) in the cytoplasm (Fig 6B, 6F, 6I and 6L) was also more than that of WT or S1027D (Fig 6, except B, F, I, and L). These results also support the idea that dephosphorylation of theSer1027 residue in ULK2 (S1027A) enhances its autophagic activity by increased association with LC3-II (Fig 6I).

Confocal microscopy of ULK2 WT, S1027A, and S1027D with endogenous Kapβ2 in HEK293 cells also supports our notion that ULK2 S1027D binds more with Kapβ2 and more is transported to the nucleus (Fig 6M), due to the fact that this protein can expose more of its PY-NLS motif to Kapβ2 because of its weak association with ATG13 or FIP200 (Fig 6C and 6G). In contrast to this, ULK2 WT or S1027A binds to Kapβ2 to a lesser extent because of the masking of the PY-NLS motif through the binding of Atg13 or FIP2000 and less is transported to the nucleus (Fig 6K or 6L). Without PKA phosphorylation, PY-NLS of ULK2 seems to be masked through the steric inhibition through its strong association with ATG13 or FIP200 (Fig 6B and 6F).

The Ser462 (^459^RRlST^463^) residue of ULK2 also matches well with the consensus sequence information [R-R-X-(S/T)-Φ], and it is possible for it to be phosphorylated by PKA (Fig 1) [21,22]. We constructed S462A and S462D mutants, transfected HEK293 cells, the performed the same experiment as with S1027A and S1027D. However, we did not observe any change in the subcellular localization of the S462A or S462D mutant or in their protein-protein interaction with Atg13 or FIP200 in the western blot pattern with a PKA phosphorylation specific antibody (data not shown), when compared with the S1027A or S1027D mutant (Fig 6). The apoptotic ability of the ULK2 S462A and S462D mutant was not changed either, compared with that of the ULK2 WT (data not shown). Thus, these results suggest that the phosphorylation of ULK2 Ser1027 (not Ser462) by PKA is the specific regulatory site for its interaction with ATG13 or FIP200 and for its subcellular localization.

In order to obtain more evidence that the subcellular localization of ULK2 is controlled by PKA phosphorylation, the endogenous ULK2 localization in HEK293 cells was observed using confocal microscopy with the treatment of a PKA activator (forskolin, FSK) or inhibitor (H89) (Fig 6N–6P) [27]. Compared with the untreated cells (Fig 6N), the cytoplasmic localization of ULK2 (Fig 6O) with H89 treatment was not dramatically changed. However, an increased nuclear localization of ULK2 (Fig 6P) with the treatment of FSK was observed, suggesting that the subcellular localization of ULK2 is changed by PKA phosphorylation. In addition, ULK2 (was phosphorylated more by PKA) was associated more with Kapβ2 in FSK-treated cells than in normal or H89-treated cells, consistent with Fig 6K–6M.

PCC between ULK2 and Kapβ2 was measured as shown in Fig 6N (0.60+/−0.12; n = 5), Fig 6O (0.56+/−0.13; n = 5), and Fig 6P (0.74+/−0.12; n = 5) using quantitative confocal microscopy. These results also support the notion that PKA phosphorylation enhances the association of ULK2 and Kapβ2. However, it is not clear at present the reason why the PKA inhibitor (H89) is less effective than FSK in the induction of a change to a nuclear localization of ULK2. The nuclear or cytoplasmic fluorescence intensity (F.I.) profile (Fig 6N–6P, right side) also confirm that the PKA-phosphorylated ULK2 shows an increased localization to the nucleus (approximately twice the amount) than to the cytoplasm (Fig 6N and 6O, right side). Using quantitative confocal microscopy, the Fn/t ratio of normal DMEM-grown HEK293 cells (49.36+/−4.90; n = 10), H89-treated cells (35.78+/−4.16; n = 10), or FSK-treated cells (52.73+/−3.65; n = 10) was also determined. Consistent with the PCC and ULK2 S1027A/D mutant results (Fig 6A–6M), the activation of PKA phosphorylation by FSK promotes ULK2 nuclear transport by an increase in ULK2 and Kapβ2 interaction. However, the inhibition of PKA phosphorylation by H89 inhibits ULK2 nuclear transport through the inhibition of ULK2 and Kapβ2 interaction (Fig 6P and 6Q).

**Fig 7:**
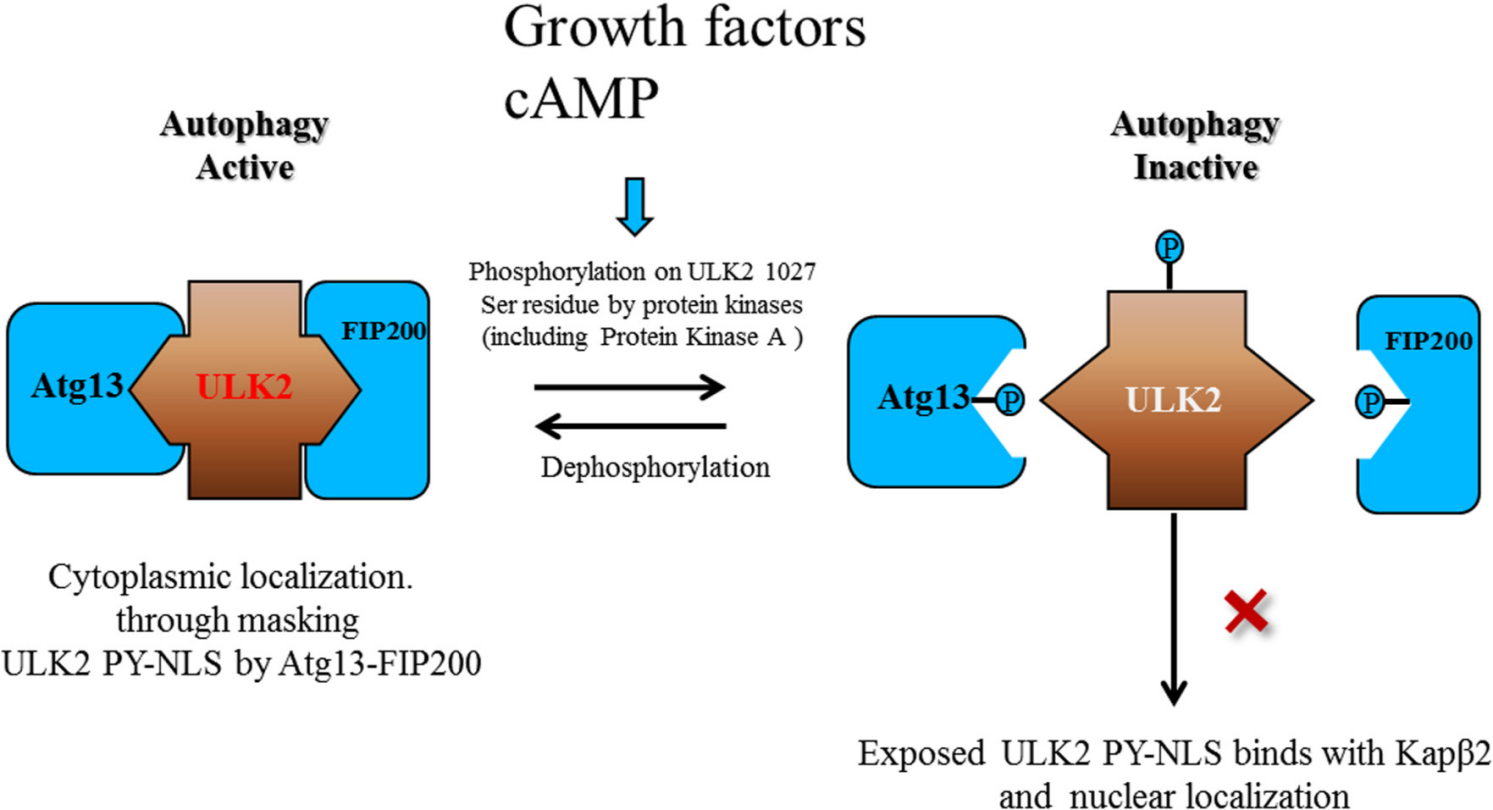
Both PY-NLS and Ser1027 residue phosphorylation in ULK2 confer a unique role in autophagy. P794A (^774^gpgfgssppGaeaapslRyvPY^795^ changed to ^774^gpGfgssppgaeaapslRyvAY^795^) or P242A (^220^qdlrmfyeKnRnotslmpSipRetsPY^243^ changed to ^220^qdlrmfyekKnRslmpSipRetsAY^243^) mutants showed an increased autophagic activity, but less serine phosphorylation. Membrane attachment and interaction with Atg13-focal adhesion kinase family-interacting protein 200 (FIP200) appears to mask the ULK2 PY NLS motif (Left side). The protein complex is likely dissociated by phosphorylation by the protein kinase (such as AMPK, mTOR1, PKA, or ULK2), consequently making the PY-NLS motif in ULK2 accessible by Kapβ2 (Right side), even though the phosphorylation sites on Atg13-FIP200 by the protein kinases are not completely characterized. Due to the fact that the PY-NLS mutant of ULK2 and WT ULK1 (“X” indicates blockade of Kapβ2 binding) are not transported to the nucleus, these two seem to be more active in autophagy than WT ULK2. The ULK2 S1027A mutant (dephosphorylated analogy form) can easily bind Atg13-FIP200 to promote cell autophagy and apoptosis. These protein associations result in its PY-NLS motif hiding through steric inhibition, resulting in the blockade of its nuclear localization by Kapβ2 (Left side). Meanwhile, the PY-NLS motif of the ULK2 S1027D mutant (PKA phosphorylated analogy form), which is free from Atg13-FIP200 association, is exposed to Kapβ2, and the mutant protein can be imported into the nuclei (Right side). The freed ULK2 S1027D mutant can induce neither cell autophagy nor apoptosis. Thus, the phosphorylation on the Ser1027 residue (RRlSA) of ULK2 by PKA seems to be a major regulatory event in its autophagic functions (see the main body text for more detail).

Therefore, we assumed that because the domain for protein-protein interaction with Atg13 or FIP200 overlaps with the ULK2 Ser1027 residue, which can be phosphorylated by PKA (Fig 1A), the subcellular localization of ULK2 S1027A (cytoplasm) or S1027D (nucleus) mutants was different (Fig 7). The PY-NLS domain of ULK2 S1027A is probably not available to Kapβ2, because Atg13 or FIP200 binds to the dephosphorylated ULK2 (S1027A). Eventually this seems to cause ULK2 S1027A to stay in the cytoplasm as an autophagically active form (Figs 4 and 6).

Even though unknown proteins interacts with the C-terminus of ULK2, the phosphorylation of the Ser1027 residue (analogous to ULK2 S1027D) by protein kinases (such as PKA) promoters the dissociation of Atg13 and FIP200 from ULK2, and the PY-NLS of ULK2 is exposed to Kapβ2 in order to facilitate transport to the nucleus (as an autophagically inactive form), otherwise the protein complex stays in the cytoplasm as an active form (Fig 7). The protein kinase that is responsible for the phosphorylation of the Ser1027 residue in ULK2 remains to be characterized, despite PKA being the best candidate kinase in our observation. We propose our working hypothesis in Fig 7. ULK2 can be transported into the nucleus through the binding of its PY-NLS motif to Kapβ2. The phosphorylation of the ULK2 Ser1027 residue may regulate this event through protein-protein interaction. Due to the fact that ULK1 does not contain a PY-NLS motif, it appears to be a cytoplasmic protein as shown in Fig 1E (similar to the ULK2 PY-NLS mutant), and its kinase activity seems to be only regulated by the upstream protein kinase phosphorylation or dephosphorylation, not by its subcellular localization.

### Effect of ULK2 localization on cell viability

We measured cell viability using FACS analysis to determine whether the subcellular localization of ULK2 influences cell viability [16]. As shown in Table 1, the FACS results indicate that cells containing the ULK2 PY-NLS or S1027A mutant (localized mainly in the cytoplasm with enhanced autophagy activity, Figs 5 and 6) showed a higher apoptosis rate than cells transfected with EGFP- ULK2 WT, P242A, S1027D (localized mainly in the nucleus), or EGFP vector alone. Instead, the promotion of nuclear localization of ULK2 by the S1027D mutation inhibited both its autophagic activity and the programed cell death in our system (Table 1 and Fig 7). Therefore, these results suggest that the subcellular localization of ULK2 seems to be related to both apoptosis and autophagy.

**Table 1:** Comparison of the effect of ULK2 WT and its mutants on cell survival.

ULK2	Rate of apoptosis (%) by FACS
WT	25 +/− 3
PY mutant (P794A)	10 +/− 4
P242A	23 +/− 4
(Vector only)	7 +/− 2
Mean value of 5 replicates	

Cells were transfected with EGFP-ULK2 WT, its PY mutants (P242A, P794A, S1027A, and S1027D) or the EGFP vector, and the rate of apoptosis was measured by FACS. The EGFP-ULK2 PY-NLS (P794A) or S1027A mutants, which are localized in the cytoplasm, promote cell apoptosis significantly more than does ULK2-WT, P242A, or S1027D, which show nuclear localization. The apoptotic effect of the ULK2 P794A and S1027A mutants was roughly half that of the ULK2 WT, P242A, or S1027D. For details, see the Materials and Methods section [16].

In summary, our data indicates that ULK2 binds to Kapβ2 via a PY-NLS motif (^774^gpGfgssppgaeaapslRyvPY^795^) in the S/P space domain resulting in nuclear localization (Figs 1–3). In addition, interaction with Kapβ2 decreases ULK2 autophagic activity by promoting ULK2 nuclear localization (Fig 7), while at the same time increasing the dissociation of Atg13 and FIP200 (Fig 6). The phosphorylation by PKA, of the ULK2 Ser1027 residue (RRlSA) in its C-terminal domain where it overlaps with the Atg13 and FIP200 association site (Fig 1A), is one of main control events in its subcellular localization and autophagic activity. Thus, we propose our working hypothesis to explain why the cytoplasmic localization of ULK2 (ULK2 PY-NLS mutant) enhances cell apoptosis and autophagic activity (Fig 7).

## Discussion

The role of ULK2 (together with ULK1) in signal transduction has been most clearly characterized in the context of autophagic kinase signaling, in which it functions as a hub protein kinase [1–11]. In the present study, we describe the *in vitro* and *in vivo* interactions of ULK2 (a member of the autophagy kinase family) with Kapβ2, which resulted in nuclear localization of ULK2, and the interaction between the two proteins was regulated by PKA phosphorylation on the Ser1024 residue of ULK2 (Fig 7). Using co-immunoprecipitation and confocal microscopy, we demonstrate that ULK2 and Kapβ2 are strongly associated (Figs 1–3). In addition, we present clear evidence that this interaction is mediated through a Kapβ2 binding motif (^774^gpGfgssppgaeaapslRyvPY^795^) in ULK2.

Most Kapβ2s directly bind the PY-NLS of their cargo [11,14–16]. Although our data suggests that the interaction between ULK2 and Kapβ2 controls the nuclear import of ULK2 (Figs 3 and 5), and reduces serine-phosphorylation and the autophagic activity of ULK2 (Figs 4 and 5), our findings raise another question: what is the function of ULK2 in the nucleus? Because the interaction between ULK2 and Kapβ2 reduces the autophagic activity of ULK2 (Fig 4) and enhances phosphorylation of its serine residues (Figs 5 and 6), the unique action of ULK2 seems to be as a shuttle messenger from the cytoplasm to the nucleus during the autophagy process (Fig 7). The Pearson's correlation coefficient between ULK2 and Kapβ2, which was 0.60+/−0.12 (Figs 1D and 6N; normal growth conditions), 0.56+/−0.13 (Fig 6O; H89 treatment), or 0.74+/-0.12 (Fig 6P; FSK treatment) as seen using quantitative confocal microscopy, also supports the idea that the association between ULK2 and Kapβ2 can be modulated by PKA phosphorylation. However, the nuclear localization of ULK2 did not change according to the cell cycle (our unpublished data). Mechanisms by which the interactions between ULK2 and Kapβ2 are controlled still need to be evaluated under physiological conditions. In addition, it remains to be determined whether the ULK2 PY-NLS mutation itself (^774^gp**G**fgssppgaeaapslRyvPY^795^ changed to ^774^gp**G**fgssppgaeaapslRyvAY^795^) affects kinase activity, regardless of protein-protein interactions with Kapβ2. It is interesting to note that one of the Kapβ2 binding motifs (^223^LrmfyeKnRslmpsipRetsPY^243^) present in ULK2 near its kinase domain (Fig 1A) is found only in ULK2 (as compared with other ULKs), suggesting that ULK2 may have a unique function in autophagy [1,4,7,9,28]. It is also of value to note that ULK2 contains a third putative PY motif (^466^padtaqtvgrRlstgssrPY^475^ **)** that does not match the consensus PY-NLS motif (R/H/KX_(2–5)_PY) [1]. We demonstrated here that ULK2 (but not ULK1) can function as a Kapβ2 substrate, resulting in localization of ULK2 to the nucleus. ULK2 may therefore play a particular autophagic role in the nucleus, however, this role is unclear. It remains to be determined whether these two motifs both contribute to the observed increase in the survival of cells containing the ULK2 PY-NLS mutant and this protein’s autophagic activity (Table 1 and Fig 5).

In comparison with the subcellular localization of ULK1 (which lacks a PY-NLS motif) in other studies [3–5], ULK2 is localized in the nucleus rather than the cytoplasm (Fig 1D and Fig 3). This may explain the autophagic activity difference between ULK1 and ULK2 (seems to be due to the difference in its subcellular localization), because the ULK2 PY-NLS mutant also showed a difference in each protein’s autophagic activity (Fig 4). However, we did not determine whether the PY-NLS mutation directly affects its serine phosphorylation (Fig 5), including Ser335 (which corresponds to Ser341 in ULK1) or Ser1032 in ULK2 (which corresponds to Thr1046 in ULK1) (Fig 1A), which is assumed to be required for its kinase activity [1,3]. Thus, it is also necessary to ascertain whether phosphorylation of Kapβ2 is necessary for the activation and/or regulation of ULK2 subcellular localization by Kapβ2 (Figs 3 and 7).

Due to the fact that the phosphorylation of Ser1027 in ULK2 by protein kinases including PKA (S1027D mutant) inhibited the autophagic activity of ULK2, and decreased sensitivity to starvation and stress by promoting nuclear localization of ULK2 (Figs 6 and 7), it seems to be that the cyclic AMP dependent cell survival signal is also transduced into the nucleus through this step.

Even though the ULK2 S1027A/D mutants showed a dramatic subcellular difference compared with WT, the subcellular localization of the ULK2 S468 A/D mutants did not change (unpublished data). The explanation for this event is the protein-protein interaction of Atg13 and FIP200 with the C-terminus of ULK2 (Fig 1A). Despite the fact that we do not exclude other possibilities (such as its structural disturbance and other modifications), it remains to be characterized why the early formation of a protein-protein complex with Atg13-FIP200 and the C-terminus of ULK2 has a tendency to recruit LC-3II and WIPI to the autophagosome rapidly, resulting in autophagy (Fig 4E and 4H, Fig 6I). However, we used these two protein appearances on the autophagosome as an indication of autophagic activity (Fig 4A–4C and 4E, Fig 6I) [8,9,19,20]. Due to the fact that the Ser1027 residue (RRlSA) of ULK2 is also conserved in the C-terminal domain of ULK1, it may be possible that ULK1 is also regulated by PKA phosphorylation, similar to ULK2, except for the nuclear localization (Fig 1). The phosphorylation of ULK2 by other protein kinases (such as mTORC1 or Akt) may also inhibit autophagic activity through its dissociation from Atg13-FIP200 or from the membrane (Figs 6 and 7), resulting in ULK2 localization to the nucleus [3–5,9,13,28]. We assumed that the ULK2 putative self-phosphorylation of Ser1032 and the autophagic activity of ULK2 are inhibited by the interaction of ULK2 with Kapβ2, similar to the phosphorylation of Ser1027 (Figs 4–7). In this case, Kapβ2 may therefore act as an antagonist of ULK2 signal transduction and autophagic function. Although the data obtained here suggests that Kapβ2 may function as a negative regulator of ULK2 signaling, the precise mechanisms underlying the subcellular localization of ULK2 require further characterization in order to gain better insight into the function of ULK2 in the autophagic signal transduction pathway [1,9]. Even though the function of autophagy in cell survival seems to be Janus, depending on the cell line and conditions, cell apoptosis and autophagy were observed relatedly in HEK293 cells, as shown in Fig 4 and Fig 6, and Table 1 [3–5,9,28]. Thus, it is also sensible to predict that cell survival signals by the PKA pathway inhibit both the autophagic activity and cell apoptosis, through the phosphorylation of the ULK2 Ser1027 residue (Figs 1A and 7 and Table 1).

The ULK2 S1027A/D mutant proteins play a dominant negative role in cell survival. While the ULK2 S1027A mutant forms more protein complexes with Atg13 and FIP200, less endogenous ULK1/2 binds with Atg13 and FIP200 (Fig 7), leading to increased apoptosis (Table 1). Reversely, the ULK2 S1027D mutant forms less protein complexes with Atg13 and FIP200, and more endogenous ULK1/2 binds with Atg13 and FIP200, contributing to decreased apoptosis (Fig 7). It may also be possible that the nuclear localized ULK2 S1027D may unexpectedly inhibit apoptotic genes. However, it remains to be characterized how the ULK2 S1027A/D mutant proteins contribute to apoptosis.

Recently, ULK1 and ULK2 were reported to have distinct functions in the regulation of lipid metabolism in adipocytes [29]. Nuclear localization of ULK2 by its PY-NLS motif is also a distinct characteristic of ULK2 that differentiates it from other ULKs (Fig 7). Furthermore, this endogenous characteristic of ULK2, which can localize to the nucleus through its PY-NLS motif, seems to be one of the reasons why the autophagic activity of ULK2 is less than that of ULK1, even though both are redundant of each other [2–5]. Thus, the nuclear localization of ULK2 by its PY-NLS motif provides a distinguishable characteristic from ULK1 for its functional regulation, even though the unique role of ULK2 in the nucleus remains to be clarified.

It is also noteworthy that ULK2 contains another putative Kapβ2 interaction site (^995^aAllleglsRilqdPa^1010^), where the binding site of Atg13-FIP200 overlaps near to the PKA phosphorylation site (S1027 residue). Therefore it seems to be the case that ULK2 phosphorylation by PKA affects both the nuclear localization and the autophagic activity of ULK2. However, even though the nuclear import of ULK2 by the interaction between its PY-NLS motif and Kapβ2 seems to be regulated by PKA phosphorylation on its S1027 residue, its mechanism of export to the nucleus is unknown. The possible nuclear export sequence (NES) motifs containing the leucine rich segments are also noticed in ULK2 with a NES consensus motif [1,30]. We are now pursuing investigations into which segment of these is the real NES motif of ULK2. Even though the NES motif of ULK2 is not yet clear, we assumed that its NES motif is also affected by Ser1027 phosphorylation, because the ULK2 S1027A or D mutant proteins have their own subcellular localization (Fig 6).

In conclusion, we demonstrated that Kapβ2 binds to ULK2 through ULK2’s PY-NLS motif (^774^gpGfgssppgaeaapslRyvPY^795^) in its S/P space domain (Fig 1A), but also to a lesser extent through another putative PY motif (^220^qdlrmfyeKnRslmpSipRetsPY^243^). Unlike ULK1, which is localized predominantly in the cytoplasm (Fig 1E), ULK2 WT is localized in the nucleus through its PY-NLS motif (Fig 1D) through shuttle protein action. The phosphorylation of Ser1027 in ULK2 by PKA decreases the autophagic activity (and the apoptosis) of ULK2, by promoting not only dissociation of Atg13 and FIP200 from ULK2 but also the nuclear localization of ULK2 (Fig 7, right). Conversely, the dephosphorylation of the Ser1027 residue of ULK2 increases its autophagic activity and apoptosis by its association with Atg13 and FIP200 and the blockade of its nuclear localization through Kapβ2 (Fig 7, left). The nuclear localization of ULK2 by its PY-NLS motif seems to be one of its distinct roles, from those of ULK1, in autophagy (Fig 1).

## Supporting Information

S1 Information S1. Data for Fig 1D and 1E. Interaction between the putative PY-NLS motif of ULK2 and Kapβ2 and the subcellular localization of ULK2 and Kapβ2. The nuclear-to-total fluorescence ratio (Fn/t). **S2. Data for** **Fig 3A, 3B and 3C**. Subcellular localization of exogenous ULK2 PY mutants. The nuclear-to-total fluorescence ratio (Fn/t). **S3. Data for** **Fig 6A, 6B and 6C**. Subcellular localization and autophagic ability of ULK2 Ser1027 mutants. The nuclear-to-total fluorescence ratio (Fn/t). **S4. Data for** **Fig 6N, 6O and 6P**. The endogenous ULK2 in HEK293 cells was visualized with a PKA inhibitor (O) and activator (P). Control (N). The nuclear or cytoplasmic fluorescence intensity profile. **S5. Data for** **Fig 6N, 6O and 6P**. The endogenous ULK2 in HEK293 cells was visualized with a PKA inhibitor (O) and activator (P). Control (N). The nuclear-to-total fluorescence ratio (Fn/t).
(XLSX)
